# Influence of catheter diameter, endotracheal tube diameter, suction pressure, and PEEP on the tracheal pressure and lung volume during endotracheal suctioning using a lung model

**DOI:** 10.1186/cc10725

**Published:** 2012-03-20

**Authors:** KJ Snijders, PE Spronk, TW Fiks, H Boon, T Ten Kleij, AA Becht, FH De Jongh

**Affiliations:** 1 Gelre Ziekenhuizen, Apeldoorn, the Netherlands; 2 Academic Medical Center, Amsterdam, the Netherlands

## Introduction

Endotracheal suctioning (ETS) is frequently performed in the ICU for clearing bronchial secretions in intubated and ventilated patients. However, research shows that subatmospheric pressures in the trachea and decreases in lung volume are measured during ETS when unfavourable parameters are chosen, causing complications (for example, atelectasis) [1,2]. The aim of this study was to investigate the influence of the parameters: area ratio 'catheter/endotracheal tube', suction pressure, type of ventilation, and positive end-expiratory pressure (PEEP) on tracheal pressure and lung volume during ETS.

## Methods

A lung model (two intersurgical balloons of 2 litres each and an artificial trachea with a 25 mm internal diameter) for spontaneous breathing and pressure-controlled ventilation (PCV) was designed. Spontaneous breathing was simulated by varying the pressure inside the chamber in which the balloons were mounted by an electronically controlled syringe. During PCV, a Servo 300 ventilator was added. An open suction system (VBM, 5 mm suction gap) was used. After insertion of the catheter, suction (pressures ranged from 20 to 65 kPa) was applied for 15 seconds during withdrawal, as used in clinical practice. During spontaneous breathing the parameters pressure and area ratio (79%, 58%, 34%, 25%, 13%) were varied, while during PCV the PEEP was varied too. Each setting was repeated three times and the mean results were used for analysis.

## Results

For spontaneous breathing (*n *= 45) the mean tracheal pressure and lung volume decreased strongly when the area ratio and/or suction pressure increased (for example, mean tracheal pressure -13 ± 1.3 cmH_2_O compared to atmospheric pressure, and lung volume -524 ± 37 ml using 20 kPa suction pressure and area ratio 0.58), the first having the greater influence. Similar results (*n *= 84) were found for PCV (for example, 22 ± 0.35 cmH_2_O and -536 ± 137 ml, using 20 kPa suction pressure, area ratio 0.58 and PEEP 20 cmH_2_O).

## Conclusion

During endotracheal suctioning the area ratio (between the catheter and the endotracheal tube) and the applied suction pressure should be minimal to avoid high pressure and lung volume losses.
